# Clusterin expression can be modulated by changes in TCF1-mediated Wnt signaling

**DOI:** 10.1186/1750-2187-2-6

**Published:** 2007-07-16

**Authors:** Troels Schepeler, Francisco Mansilla, Lise L Christensen, Torben F Ørntoft, Claus L Andersen

**Affiliations:** 1 Molecular Diagnostic Laboratory, Department of Clinical Biochemistry, Aarhus University Hospital, Aarhus, Denmark

## Abstract

**Background:**

Clusterin (CLU) is an enigmatic molecule associated with various physiological processes and disease states. Different modes of cellular stress lead to increased CLU levels, and additionally numerous growth factors and cytokines affect the expression of the CLU gene. APC and c-MYC, both intimately linked to the Wnt signaling pathway have previously been shown to influence CLU levels, and we therefore investigated if changes in Wnt signaling activity *in vitro *could regulate the expression of one, or more, of several CLU mRNA and protein variants.

**Results:**

Over-expression of the cytoplasmic domain of E-cadherin tagged with GFP was used to abrogate Wnt signaling activity in LS174T and HCT116 colon carcinoma cells. This fusion construct sequestered signaling competent β-catenin whereby Wnt signaling was abrogated, and consequently cytoplasmic CLU protein levels increased as demonstrated by immunofluorescence. To determine which branch of the Wnt pathway was mediating the CLU response, we over-expressed dominant negative (dn) TCF1 and TCF4 transcription factors in stably transfected LS174T cells. We observed both intra- and extracellular levels of CLU protein to be induced by dnTCF1 but not dnTCF4. Subsequent analysis of the expression levels of three CLU mRNA variants by real time RT-PCR revealed only one CLU mRNA variant to be responsive to dnTCF1 over-expression. 5'-end RACE indicated that this CLU mRNA variant was shorter at the 5'-end than previously reported, and accordingly the translated protein was predicted to be shorter at the N-terminus and destined to the secretory pathway which fit our observations. Examination of the immediate expression kinetics of CLU after dnTCF1 over-expression using real time RT-PCR indicated that CLU might be a secondary Wnt target.

**Conclusion:**

In conclusion, we have demonstrated that the Wnt signaling pathway specifically regulates one out of three CLU mRNA variants via TCF1. This CLU transcript is shorter at the 5' end than reported by the RefSeq database, and produces the intracellular 60 kDa CLU protein isoform which is secreted as a ~80 kDa protein after post-translational processing.

## Background

The clusterin (CLU) protein was first discovered more than 25 years ago in rat testis fluid because of its ability to facilitate clustering of a variety of cell types in culture[1]. Since then, homologues of the broadly expressed CLU gene have been identified in several species and CLU proteins have been found in most mammalian body fluids[2]. CLU is an enigmatic molecule, implicated in diverse biological processes, and has additionally been associated with opposing functions in regard to apoptosis. Accumulating evidence from several studies suggests that the pro- and antiapoptotic functions may be related to nuclear and secreted protein isoforms, respectively[3]. The secreted form of CLU is a glycosylated protein of 70–80 kDa that consists of two chains held together by five disulfide bonds, and consequently it appears as a ~40 kDa smear on immunoblots from reducing SDS-PAGE. Its intracellular pre-curser form of 60 kDa may also exhibit an antiapoptotic function[4]. The proapoptotic CLU variant is a 50–55 kDa protein which accumulates in the nucleus of apoptotic cells[3]. How these different CLU protein variants are produced from the CLU gene is poorly understood, although it has been speculated that nuclear CLU results from an alternative splice event skipping exon 2 from the main CLU transcript otherwise translated into secreted CLU[5].

CLU is recognized as being a stress-inducible gene, thus responding to a variety of general stress stimuli such as oxidative stress, ionizing radiation and heatshock. In addition, several cytokines and growth factors either promote or suppress CLU expression *in vitro*[2]. Numerous functional *cis*-elements and *trans*-factors have been identified which are responsible for regulating CLU expression under different conditions *in vitro*. *Trans*-factors which have been shown to interact with the CLU promoter, and regulate its activity, include Egr-1[6], members from the AP-1 complex[7], HSF1/2[8], Cdx1/2[9], and B-MYB[10]. Hence, many *cis*/*trans*-factors have been identified which may be responsible for the complex tissue-specific control of the gene. The complexity of CLU regulation is also illustrated by the observations that identical growth factors may elicit different CLU responses depending on the cell type of exposed cells, and whether cells are grown as mono- or mixed cultures *in vitro*[2]. In contrast to the avian CLU gene whose expression can be driven by two alternative promoters[11], CLU expression in mammals has traditionally been thought to be controlled by one promoter only. However, several novel CLU mRNA variants have recently been identified in human colon and prostate cells, and two of these transcripts were demonstrated to be differentially regulated by androgens in prostate cells[12,13]. Considering the recent discovery of these CLU transcripts little is known about their biological relevance and regulation, including whether transcription of each mRNA species is initiated from the same promoter or perhaps different promoters. Furthermore, it is also unclear how they each relate to the different CLU isoforms observed at the protein level. Investigating these issues is important for understanding CLU regulation and function. Over-expression of oncogenes B-MYB and c-MYC, has been reported to induce and repress CLU, respectively[10,14]. Of note, c-MYC is recognized as being a target of the Wnt signaling pathway[15]. Another Wnt component, APC, lead to elevated CLU levels when being over-expressed in a null APC-/- human colon cancer cell line[16]. Collectively, these studies made us speculate that one, or more, of the CLU mRNA variants might be regulated by Wnt signaling. Accordingly, we used a model system based on human colon adenocarcinoma cells to investigate whether CLU expression could be regulated by changes in Wnt signaling. Indeed, we show that by abrogating the otherwise constitutively activated Wnt pathway in these cells, CLU protein levels are up-regulated. Concomitantly, we observed increased levels of one out of three CLU mRNA variants, previously identified in colonic tissue. Induction of CLU is further demonstrated to be mediated by the transcription factor, TCF1.

## Results

### Sequestration of β-catenin increases CLU protein levels in colon carcinoma cells

To determine if Clusterin protein levels are affected by alterations in Wnt signaling activity, we transiently transfected the colon carcinoma cell lines, LS174T and HCT116, with a fusion construct consisting of GFP fused to the cytoplasmic domain of E-cadherin. The encoded fusion protein efficiently sequesters signaling competent β-catenin, thus abrogating the Wnt signaling pathway which is otherwise constitutively activated in these cell lines due to activating mutations in β-catenin.

**Figure 1:**
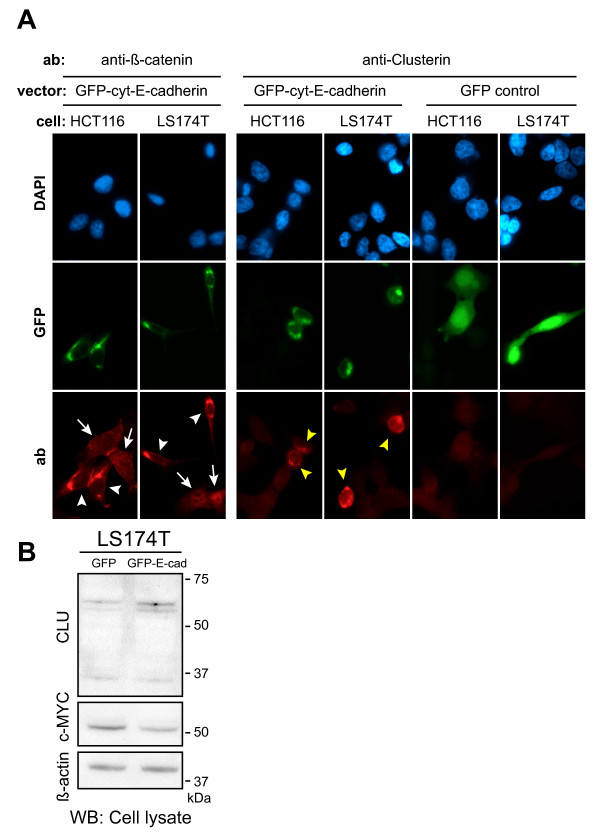
**Sequestration of β-catenin by GFP-tagged E-cadherin lead to up-regulation of CLU levels in colon carcinoma cells**. Changes in CLU protein levels were investigated in response to over-expression of GFP tagged E-cadherin and GFP as control. **(A) **Immunofluorescence was used to show the effects of transient over-expression of GFP tagged E-cadherin, which binds β-catenin, in LS174T and HCT116 colon carcinoma cell lines. In the left panel it is shown that the GFP-cyt-E-cadherin fusion protein efficiently sequesters β-catenin from the nucleus whereupon Wnt signaling is abrogated. In transfected cells (white arrow heads) β-catenin has a perinuclear localization contrasting untransfected cells (white arrows) where it is uniformly distributed. Shown in the right panel is the up-regulation of cytoplasmic CLU protein which follows the abrogation of Wnt signaling induced by the GFP-cyt-E-cadherin fusion protein. CLU is up-regulated in GFP-cyt-E-cadherin transfected cells (yellow arrow heads) but not in untransfected cells nor in cells transfected with GFP alone. **(B) **Western blot with cell lysates from LS174T cells 24 hr after transient transfection with GFP-cyt-E-cadherin and GFP as a control. β-actin was used as a loading control. CLU protein levels are up-regulated and c-MYC protein levels are down-regulated as a consequence of over-expressing GFP-cyt-E-cadherin but not GFP alone. WB: western blot, ab: Antibody.

To validate that the fusion protein indeed sequestered β-catenin, we first performed immunofluorescence using an anti-β-catenin antibody. As expected, over-expression of the GFP-cyt-E-cadherin construct resulted in a strong decrease of nuclear β-catenin staining in both cell lines, which instead co-localized with the GFP-cyt-E-cadherin fusion in a perinuclear pattern (white arrow heads; Fig. 1A). When staining transfected cells using an anti-CLU antibody, CLU was found to be predominantly up-regulated in GFP-cyt-E-cadherin transfected cells relative to GFP-transfected cells (and non-transfected cells), and exhibited a cytoplasmic localization (yellow arrow heads; Fig. 1A, and Additional file 1). In GFP-transfected and non-transfected cells, the CLU signal was close to background and CLU staining, if any, was confined to the cytoplasm. In agreement with these results western blotting demonstrated that CLU was up-regulated in LS174T cells transiently transfected with GFP-cyt-E-cadherin relative to the GFP control transfectants (Fig. 1B). To make sure that the GFP-cyt-E-cadherin affected the expression levels of known Wnt target genes western blotting was used to detect changes in the levels of the well-known Wnt target, c-MYC. In accordance with c-MYC being positively regulated by Wnt signaling[15], c-MYC protein levels were reduced upon expression of GFP-cyt-E-cadherin confirming that the GFP-cyt-E-cadherin construct had a functional impact on the expression of Wnt target genes. The up-regulation of CLU (and down-regulation of c-MYC) detected by western blotting was modest, which is possibly attributable to the combination of a moderate transfection efficiency (~30%) and that only a subpopulation of transfected cells up-regulate CLU (see Additional file 1).

Altogether the data showed that inhibition of Wnt signaling by sequestration of β-catenin led to increased CLU levels.

### Wnt regulation of CLU is mediated by TCF1

**Figure 2:**
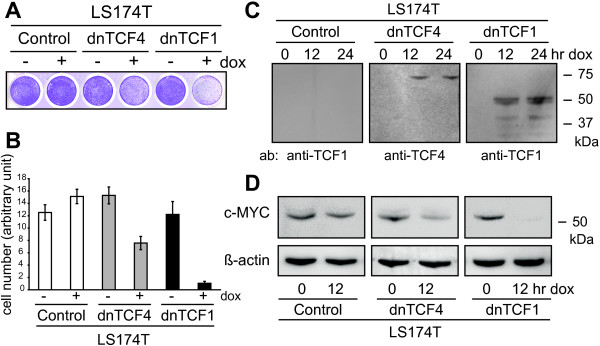
**Evaluation of dnTCF-Wnt-model system: Expression of exogenous gene products and phenotypic change**. Doxycycline-inducible dominant negative (dn) versions of TCF1 and TCF4 factors, the effectors of the Wnt pathway, were over-expressed in stably transfected LS174T derived cell lines. **(A) **Proliferation was halted in LS174T derived cell lines after induction of both dnTCFs. This was visualised by methyl violet staining of cell cultures after 5 days of induction. **(B) **Similar results were obtained by manually counting cells in a haemocytometer after cells had been cultured in the presence (+) or absence (-) of induction for 4 days. Data are presented as the mean ± standard deviation from 3 separate experiments. **(C) **Induction of exogenous gene products, dnTCFs, was detected by western blot with cell lysates from LS174T derived cell lines 0, 12, and 24 hr after induction. Control cells are LS174T cells without any dnTCF expression vector. **(D) **Both dnTCF1 and dnTCF4 abrogate β-catenin/TCF driven transcription of the Wnt-target gene, c-MYC, as analyzed by western blot with cell lysates from LS174T derived cell lines 0 and 12 hr after induction.

The outcome of the Wnt signaling pathway is ultimately determined by its most downstream effectors; members from the T-cell factor (TCF)/lymphoid enhancer factor (LEF) family. To elucidate which TCF-branch of the Wnt signaling pathway was responsibly for the observed changes in CLU protein levels, we used LS174T cells stably transfected with either doxycycline-inducible dominant negative (dn) TCF1 or TCF4 factors. As a first step we evaluated these model systems. Western blotting confirmed that both dnTCF1 and dnTCF4 proteins were strongly induced upon addition of doxycycline (Fig. 2C). In agreement with previous reports, expression of either dnTCF resultated in a strong decrease in cell growth rate as verified by two independent proliferation assays (Fig. 2A,B). Having confirmed the induction of exogenous dnTCFs and the accompanying growth inhibitory phenotype, we also wanted to make sure that the expression levels of transcriptional Wnt targets were affected. Accordingly, we used western blotting to detect changes in the levels of the Wnt target, c-MYC, which were shown to be drastically reduced upon expression of either dnTCF (Fig. 2D). Collectively, these observations suggested the stably transfected LS174T cell lines to be a reliable Wnt model system.

**Figure 3:**
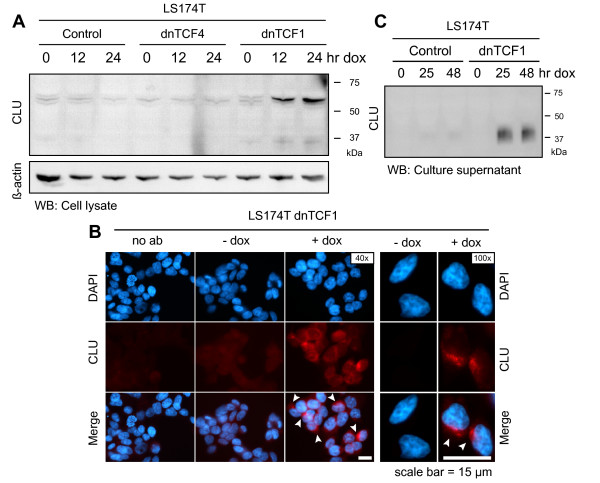
**>Intra- and extracellular CLU protein levels are increased specifically in response to dnTCF1 over-expression**. Changes in CLU protein levels were investigated in response to over-expression of dnTCF1 and dnTCF4 in stably transfected LS174T derived cell lines. **(A) **Western blot with cell lysates from LS174T derived cell lines 0, 12, and 24 hr after induction. β-actin was used as a loading control. CLU protein levels are up-regulated after induction of dnTCF1, but not dnTCF4, and appear as a strong 60 kDa band and a faint ~40 kDa band. **(B) **Immunofluorescence in LS174T dnTCF1 cells, fixed 24 hr after either induction (+dox) or no induction (-dox). Intracellular CLU levels are up-regulated in response to induction of dnTCF1. The subcellular localization of up-regulated CLU is mainly cytoplasmic (white arrows). No (primary) antibody control is also shown. **(C) **Over-expression of dnTCF1 in LS174T cells also led to increased levels of extracellular CLU as demonstrated by western blot with acetone-precipitated total proteins from culture supernatant 0, 25, and 48 hr after induction. CLU appears as a ~40 kDa smear which is consistent with the secreted form of CLU being a cleaved glycosylated protein that consists of two chains of similar molecular weight, held together by several disulfide bonds.

Western blotting was used to detect changes in CLU protein levels upon expression of either dnTCF. Interestingly, only over-expression of dnTCF1, but not dnTCF4, lead to increased CLU protein levels (Fig. 3A). No increase in CLU protein levels was evident in control cells not stably transfected with dnTCF-vectors but exposed to doxycycline. To exclude the possibility that loss of integrity of the CLU locus was the reason why CLU was not induced after dnTCF4 over-expression, we sequenced the coding regions of the CLU gene in the stably transfected LS174T dnTCF4 cell line as well as the dnTCF1 and control cell lines. No mutations were identified, only a silent C/T SNP in exon 5 of all three LS174T derived cell lines (data not shown), thus strengthening the conclusion that CLU was exclusively regulated by TCF1. The induced CLU protein species appeared as a distinct band of 60 kDa and a faint band of ~40 kDa (Fig. 3A). CLU exists as different protein isoforms which are reported to localize differently both intra- and extracellularly. The 60 kDa CLU isoform has consistently been reported to exhibit a cytoplasmic localization pattern which is exactly what we observed in our transient transfection experiments using GFP-cyt-E-cadherin (Fig. 1A). To confirm that CLU exhibited a similar subcellular distribution in dnTCF1 over-expressing cells, we performed immunofluorescence using an anti-CLU antibody. In agreement with our previous results the induced CLU proteins were found to be localized in the cytoplasm (Fig. 3B).

### Wnt signaling regulates the secreted CLU protein isoform

The 60 kDa CLU isoform can be converted to a ~80 kDa heterodimer isoform through glycosylation and proteolytic cleavage[17]. Consequently, this mature CLU isoform appears as a ~40 kDa smear on reducing SDS-PAGE immunoblots[2,3]. In contrast to the cytoplasmic 60 kDa form, this latter CLU protein species is secreted by the cells and can thus be found in the extracellular milieu. However, under certain stress conditions, CLU can be diverted from its normal secretory pathway and reach the cytosol by means of retrotranslocatio[18]. To investigate whether the CLU species induced by abrogated Wnt signaling was indeed secreted, western blotting was used to detect secreted CLU proteins in the culture medium at various time points after induction of dnTCF1. Our analysis revealed that CLU accumulated in the culture medium after induction of dnTCF1 (Fig. 3C). This was not the case for control cells. This implies that the up-regulation of the intracellular pre-curser form of CLU, 60 kDa, is followed by maturation and secretion of the mature CLU form. Accordingly, secreted CLU appeared as a ~40 kDa smear on the immunoblots. Not much, if any, of the ~40 kDa smear band was observed to accumulate intracellularly (Fig. 3A). This may be explained by a high secretion rate of CLU, which would keep the intracellular pool of mature CLU protein low, and has been observed to occur rapidly in human liver carcinoma cells (HepG2) with half-times of 30–35 min[17,19].

### dnTCF over-expression increase cell death rate in LS174T cells

**Figure 4:**
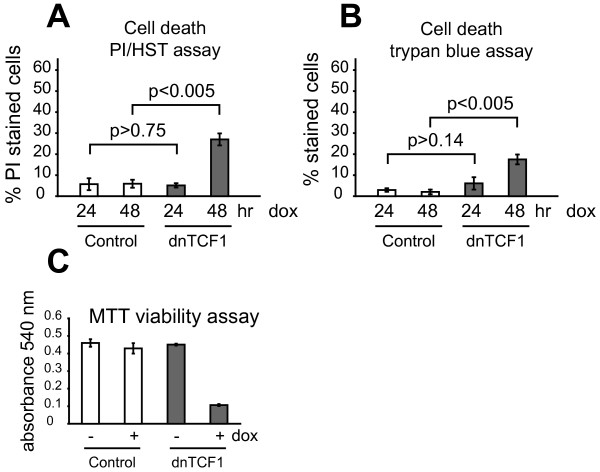
**Over-expression of dnTCF1 lead to reduced viability and increased cell death rate in LS174T cells**. Over-expression of dnTCF1 significantly increases the rate of cell death in LS174T cells 48 hr after induction (p < 0.005, student's t-test). This was determined using two cell death assays based on either trypan blue dye or DNA-intercalating dyes, propidium iodide (PI) and hoechst 342 (HST). LS174T control and dnTCF1 cell lines were cultured in the presence of induction. After 24 hr and 48 hr, floating and attached cells were stained with the respective dyes. Data from both assays are presented as the mean ± standard deviation from 3 separate experiments. **(A) **PI/HST assay: Cells were stained with PI (stains apoptotic/necrotic cells) and HST (stains viable cells). Percentage of PI stained cells relative to total cell number reflect the extent of cell death. **(B) **Trypan blue dye exclusion assay: Cells were stained with the trypan blue dye. Percentage of trypan blue stained cells (stains dead cells) relative to total cell number reflect extent of cell death. **(C) **MTT viability assay: The cells' capability to metabollically convert the MTT substrate was quantified, 48 hr after either induction (+dox) or no induction (-dox), by solubilizing MTT formazan crystals and performing spectrophotometry at 540 nm. Cell viability, which is proportional to the absorbance at 540 nm, decreases in dnTCF1 over-expressing LS174T cells relative to control cells.

Increased CLU levels have often been associated with necrotic tissues, and apoptotic cells in particular, although it is still unclear whether CLU is a cause or consequence of dying cells[2,3]. Abrogation of Wnt signaling in colon carcinoma cells is known to result in a robust growth arrest and may further enhance cell death rate[16,20]. To investigate whether the observed changes of CLU expression was simply a secondary effect of increased cell death rate we used two independent cell death assays to quantify dying cells. We observed no increases in cell death rate within 24 hr after induction of dnTCF1 in LS174T cells (Fig. 4A,B) which is in agreement with a previous study that obtained similar results within 20 hr of induction[20]. However, at 48 hr after induction a significant increase in cell death rate was observed (p < 0.005; Fig. 4A,B). Accordingly, a reduction in cell viability was also observed after 48 hr using a MTT assay which measured cells ability to metabolically convert the MTT substrate (Fig. 4C). Considering CLU is up-regulated already after 12 hr (Fig. 3A), and increased cell death rate is not observed until 48 hr after induction (Fig. 4A,B), it does not appear as if CLU is a secondary effect of cell death. Accordingly, the apoptosis-associated nuclear 50–55 kDa CLU protein variant (nCLU) was apparently not induced (Fig. 3A,B) which would have been expected had CLU been induced by dying cells.

### Characterization of the 5' end of the NM_001831.2 mRNA variant

**Figure 6:**
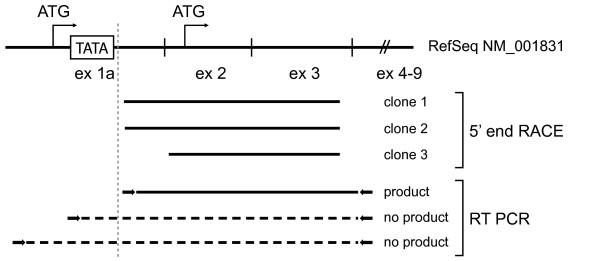
**Characterization of the 5'-UTR of the CLU34 transcript by 5'-end RACE and RT-PCR**. A colon adenocarcinoma cDNA library was used for 5'-end rapid amplification of cDNA ends (RACE) to determine the sequence of the extreme 5'-end of CLU34 mRNA. None of the three cloned 5'-end RACE fragments or the RT PCR analysis supported the most 5' extreme end of the CLU34 transcript containing the putative TATA-box as suggested by the RefSeq database. The 5'-end RACE results were consistent with results from RT-PCR reactions which only yielded an amplification product if the sense primer was placed downstream of the putative TATA-box.

Previously we have demonstrated that three different CLU mRNA variants are expressed in cancerous and normal colonic epithelial cells[12]. One of these variants corresponded to the NCBI RefSeq sequence NM_001831.2. Curiously, a closer inspection of the first exon in the NM_001831.2 transcript revealed the sequence to contain the putative TATA-box sequence previously reported for this transcript[21]. This was unexpected considering the transcription machinery involving RNA polymerase II usually initiates transcription downstream from the TATA-box which is centered between positions -30 and -100. By consulting the CLU unigene cluster (Hs. 436657) through the UCSC genome browser[22] only one full-length cDNA out of 90 supported the extreme 5' end covering the TATA-box sequence. Additionally, we were unable to generate RT-products when placing the sense primer upstream of the TATA-box sequence. Collectively, these observations made us question whether the NM_001831.2 mRNA sequence obtained from the RefSeq database was valid. Specifically, we suspected the transcript to be shorter at the 5' end and thus not contain the TATA-box motif. To resolve this issue, we performed 5' rapid amplification of cDNA ends (RACE) using a colon adenocarcinoma adaptor-ligated cDNA library. The 5' end of the transcript was determined by sequencing three cloned PCR fragments. Neither cloned fragment supported the extreme 5' end of the NM_001831.2 sequence overlapping the TATA-box (Fig. 6). In conclusion, it appears as if the NM_001831.2 transcript is not expressed in colonic cells, rather a similar but shorter transcript is expressed (hereinafter referred to as CLU34). Importantly, the proteins predicted from the two sequences differ (see below).

### In silico analysis of CLU mRNA variants

**Figure 5:**
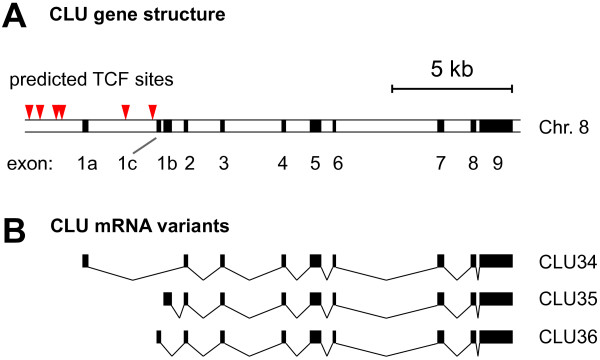
**Genomic structure of CLU locus and exon-composition of CLU mRNA variants expressed in colonic cells**. **(A) **Diagram depicts the genomic location of all CLU exons on chromosome 8 and potential TCF-binding sites (red arrows) predicted by the MatInspector software. **(B) **Exonic structure of CLU mRNA variants.

In addition to CLU34, two other variants, referred to as CLU35 and CLU36, have also been identified in colon tissue [12]. These transcripts all consist of 9 exons and only differ in regard to their first exon (Fig. 5B). Which one of these transcripts is responsible for the individual CLU protein isoforms is at present still unclear.

Using the largest open reading frame (ORF), we predicted the CLU34 and CLU35 mRNA isoforms to produce identical proteins of 449 amino acids (AAs). In contrast, the CLU36 transcript produces a hypothetical protein of 460 AAs. The subcellular localization of these proteins was predicted[23] to be nuclear in the case of CLU36, whereas the proteins produced from the CLU34/35 transcripts were secreted. Of notice, had CLU34 shared its 5' end with the NM_001831.2 RefSeq variant then a predicted nuclear protein of 501 AAs would be produced with expected distinct biological functions.

To summarize, the *in silico *predictions suggested that CLU34 and CLU35 might, individually or in combination, be responsible for the generation of cytoplasmic and secreted CLU proteins.

### TCF1-mediated Wnt signaling specifically regulates the CLU34 mRNA variant

To determine if any of these CLU mRNA species was responsive to alterations in Wnt signaling, in which case they would be likely templates for the production of the 60 kDa/~40 kDa CLU proteins, we quantified the relative expression levels of each mRNA variant by real time RT-PCR at various time points after induction of dnTCF1.

**Figure 7:**
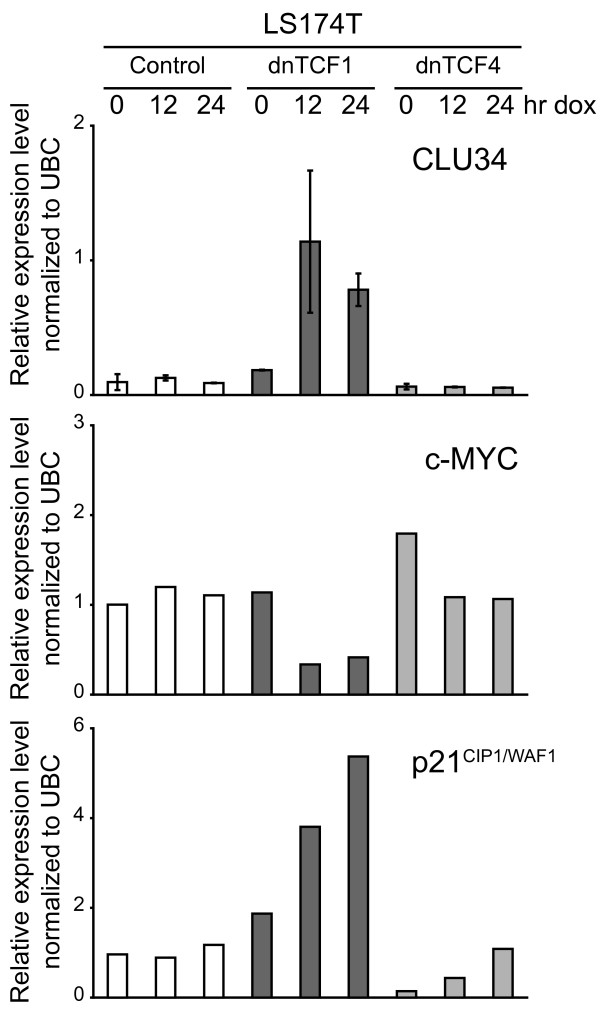
**Alterations of CLU, p21, and c-MYC mRNA levels after induction of dnTCFs in LS174T cells**. The expression levels of various transcripts were monitored by real time RT-PCR after induction of dnTCFs in LS174T derived cell lines. Expression levels were normalized to the Ubiquitin C (UBC) transcript. The CLU34 mRNA variant is specifically up-regulated in response to dnTCF1 over-expression in LS174T cells. Data are presented as the mean ± standard deviation from 2 separate experiments with each experiment consisting of the mean value of 3 independent determinations. c-MYC mRNA levels decrease, whereas p21^CIP1/WAF1 ^mRNA levels increase after induction of both dnTCFs.

Neither the CLU35 nor the CLU36 mRNA variants were responsive to alterations in Wnt signaling. In fact, they were expressed at very low levels, often below the detection limit of the real time RT-PCR assay (data not shown). In contrast, CLU34 mRNA baseline expression levels were higher, and intriguingly, this particular mRNA variant was strongly up-regulated as a response to over-expression of dnTCF1 (Fig. 7). These observations suggested CLU34 mRNA to be the template for the production of the the 60 kDa/~40 kDa CLU proteins.

To verify that these transcriptional responses were specific for dnTCF1, and not dnTCF4, we used real time RT-PCR to measure the expression levels of each CLU mRNA variant in response to dnTCF4 over-expression. As expected, none of the CLU mRNA variants responded to the induction of dnTCF4 (Fig. 7), and in agreement with our previous observations both CLU35 and CLU36 were expressed at very low levels, often below the detection limit of the real time RT-PCR assay (data not shown). Thus, CLU34 is specifically regulated by TCF1 in LS174T cells. In addition, over-expression of both dnTCF1 and dnTCF4 lead to a marked reduction of c-MYC mRNA levels which is consistent with c-MYC also being down-regulated at the protein level (Fig. 2D). Also, in agreement with previous reports, p21^CIP1/WAF1 ^mRNA levels increased as a consequence of abrogated Wnt signaling by either dnTCF[20].

To investigate whether the extremely low expression levels of CLU35 and CLU36 were a characteristic unique to our model system, we screened a large panel of cell lines for baseline expression levels of these transcripts. Besides a variety of colon carcinoma cell lines, we also included a number of prostate carcinoma cell lines in our analysis since CLU has been reported to be up-regulated in this cancer type[24,25]. In all 21 cell lines investigated, including LS174T and HCT116, both CLU35 and CLU36 were expressed at very low levels (see Additional file 2). In several cell lines the transcripts were expressed below the detection limit of the real time RT-PCR assay, and their expression levels did not correlate with that of CLU34 which was expressed at varying levels across the entire panel of cell lines (see Additional file 2). It therefore appears as if the expression of the CLU35 and CLU36 transcripts are regulated by a promoter different from the promoter controlling CLU34 expression, and that the former is only minimally activated and may require specific signals such as androgens to get stimulated[13]. This notion fits the observation that CLU35 can be specifically up-regulated by androgens and that the responsive elements are located within the first 1200 bp of the CLU35 transcriptional initiation site[13]. This genomic region is located approximately 2000 bp downstream from the CLU34 promoter/initiation site (Fig. 5).

### Expression kinetics of CLU34 in response to over-expression of dnTCF1

While the expression of direct Wnt target genes is activated by binding of TCF transcription factors to specific sequence motifs in their promoters, the expression of indirect target genes are regulated via transcription regulators, which are targets of the Wnt pathway. For example, the direct Wnt target gene c-MYC, represses the expression of the indirect target p21^CIP1/WAF1 ^by binding to the transcriptional activator MIZ1 at the p21^CIP1/WAF1 ^promoter[20].

To get an indication of whether CLU was a direct or indirect Wnt target, we searched the genomic DNA sequences upstream of each unique CLU exon 1 for TCF binding motives as these would be a prerequisite for direct regulation of CLU by the Wnt pathway. Our analysis using the MatInspector software[26] revealed a cluster of four potential TCF binding sites upstream of exon 1a (CLU34). Two additional potential TCF binding sites were localized downstream from exon 1a in the DNA region spanning exon 1a and exon 1c (Fig. 5A).

**Figure 8:**
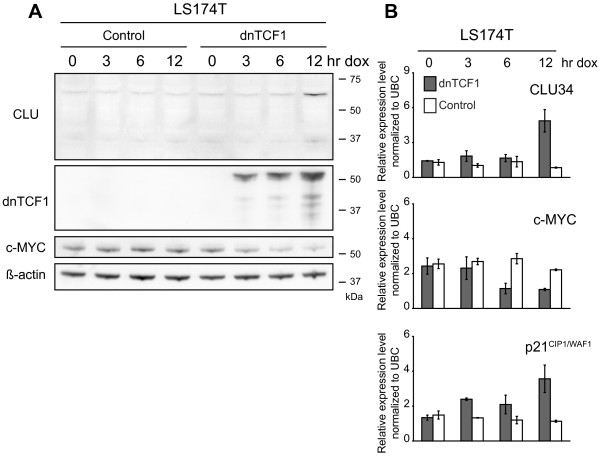
**Expression kinetics of CLU mRNA and protein levels after induction of dnTCF1 in LS174T cells**. Expression kinetics of CLU mRNA and protein levels were studied 0, 3, 6, and 12 hr after induction of dnTCF1 in LS714T cells. c-MYC and p21^CIP1/WAF1 ^mRNA levels were also monitored. **(A) **Western blot with cell lysates from LS174T cells 0, 3, 6, and 12 hr after induction. CLU protein levels do not increase until 12 hr after induction. Exogenous dnTCF1 proteins are induced as early as 3 hr after induction, and c-MYC protein levels decrease in parallel. **(B) **Real time RT-PCR shows that CLU34 and p21^CIP1/WAF1 ^mRNA levels do not increase until 12 hr after induction. c-MYC mRNA levels decrease 6 hr after induction. Data are presented as the mean ± standard deviation from 2 separate experiments with each experiment consisting of the mean value of 3 independent determinations.

We next investigated the immediate expression kinetics of CLU by measuring both CLU protein and mRNA levels at several time points within a 12 hr period after induction of dnTCF1. We rationalized that the earlier a rise in CLU levels could be demonstrated, the more likely it was that CLU was directly regulated by Wnt signaling via TCF1. Western blotting showed that dnTCF1 proteins could be detected as early as 3 hr after addition of doxycycline (Fig. 8A). CLU levels, however, did not increase until 12 hr after addition of doxycycline. In comparison, the direct Wnt target, c-MYC, was down-regulated already after 6 hr. Expression kinetics at the transcriptional level for both CLU and c-MYC were highly similar to that seen at the protein level (Fig. 8B). These observations suggested that CLU might be an indirect target of Wnt signaling, although it cannot be ruled out that (dn)TCF1s do bind directly to the CLU promoter but secondary changes are required before CLU can be induced.

We used real time RT-PCR to see if the transcriptional response of the secondary Wnt target, p21^CIP1/WAF1^, matched that of CLU[20]. This was indeed the case as p21^CIP1/WAF1 ^mRNA levels did not increase markedly until 12 hr after addition of doxycycline (Fig. 8B). In conclusion, these data points towards CLU as being a secondary target of the Wnt signaling branch mediated by TCF1 although the specific *trans*-factors responsible for CLU induction remain to be experimentally identified in future studies.

## Discussion

Here, we demonstrate that abrogation of the Wnt signaling pathway in colon carcinoma cells lead to up-regulation of CLU. By further dissecting the molecular signaling cascade responsible for increased CLU levels, we find that changes in CLU expression are specifically controlled by the branch of the Wnt pathway mediated by the transcription factor TCF1.

Our finding, that CLU expression can be regulated by the Wnt signaling pathway, add to the plethora of stimuli which can regulate CLU levels. The CLU promoter has already been shown to be responsive to a variety of external stress-inducing agents such as oxidative stress, ionizing radiation, heat shock, and chemotherapeutics[2]. In addition several types of growth factors including TGF-β, NGF, EGF, PDGF, βFGF, and interleukin-1,-2 and -6 have been demonstrated to influence CLU expression in cultured cells[2]. Although some of these signals may ultimately converge on identical downstream effectors directly responsible for regulating CLU promoter activity, several unique *trans*- and *cis*-factors have been demonstrated to influence CLU levels[2,3]. Thus, CLU expression can be modulated by many factors and their impact on CLU levels may in some cases be context dependent. For example, TGF-β and EGF treatment elevate CLU levels in some cell types whereas the very same growth factors suppress CLU expression in others[2]. Also, interleukin-6 induced CLU expression in hepatoma cells (HepG2) whereas CLU was down-regulated in rat glial cultures exposed to the same cytokine[27,28].

The Wnt signaling pathway is known to be involved in the development of colorectal cancer and is also of major importance in colon physiology[29]. In particular, this pathway controls the maintenance of colon tissue architecture by controlling epithelial cell behaviour in the crypts of Lieberkühn. The pathway is activated in epithelial cells at the crypt bottom and decreases in a gradient toward the crypt top. In colonic epithelial cells, it may therefore be expected that CLU is expressed in an inverse pattern to that of Wnt signaling. This, however, does not seem to be the case in the adult human colon as it has previously been reported that CLU only stains a small fraction of scattered colonic epithelial cells with neuroendocrine differentiation[12]. In this tissue it therefore appears as if the Wnt pathway is not the dominant force controlling CLU expression. Rather, CLU expression is likely the result of a complicated interplay between various pathways. The notion that the complexity of CLU regulation *in vivo *is not always being fully recapitulated by *in vitro *model systems, was also recently emphasized by Patel et. al who unexpectedly observed decreased CLU levels with rat donor age in glial cultures, despite CLU *in vivo *shows an opposite adult age trend: CLU expression increases during aging in select brain regions and in neurodegenerative disorders[27]. Although Wnt signaling may not be the primary regulator of CLU expression in human colonic epithelial cells, other studies indicate that this might be the case in epithelia of the murine intestine. Lars E. French and colleagues used in situ hybridization (ISH) to investigate CLU expression during murine embryogenesis, and found CLU to be weakly expressed in dividing epithelial cells at the base of the villi in the small intestine but strongly expressed in more differentiated cells that line the rest of the villus[30]. Similarly, Suh E. and colleagues used immunohistochemistry (IHC) to show that epithelia of the developing murine intestine was positive for CLU protein staining, and exhibited a gradient of activity with higher expression in the villus compartment than in the crypts[9]. At least in the developing murine small intestine, it may therefore be that Wnt signaling contributes to modulate CLU expression in epithelial cells.

Although we identified which TCF factor was responsible for CLU up-regulation, namely TCF1, it remains to be demonstrated whether TCF1, or other downstream effectors, bind directly to the CLU promoter and regulate its activity. A previous study reported the Wnt target, c-MYC, to repress CLU expression in murine colonocytes although no direct interaction between c-MYC and the CLU promoter was demonstrated[14]. In agreement with these observations we observed a decrease in c-MYC levels before CLU was induced upon over-expression of dnTCF1 in LS174T cells. However, considering that c-MYC levels also dropped when we over-expressed dnTCF4 and CLU levels remained unchanged, we do not believe c-MYC to regulate CLU levels in our model system. Notably, other investigators found no changes in CLU levels in murine fibroblast cells stably transfected with a c-MYC construct compared to mock transfected cells[31]. Therefore, other factors might be more likely to regulate CLU in our model system, possibly, AP-1 or Egr-1 transcription factors which can bind directly to the CLU promoter[6,7] and have been demonstrated to be suppressed by Wnt signaling[32].

It has become clear that the CLU gene encode several mRNA variants. At least three CLU mRNA variants are expressed in colon tissue, CLU34, CLU35, and CLU36. It has previously been reported that only one of these variants, CLU35, was down-regulated in human colorectal tumors samples compared to normal tissue indicating that the CLU mRNA variants may be differentially regulated[12]. This view was supported by a recent study by Cochrane et al. who used real time PCR to demonstrate that two CLU transcripts could be differentially regulated in prostate cells by androgens[13]. Interestingly, our results indicate that the Wnt signaling pathway, via TCF1, specifically regulate the expression levels of CLU34. Our 5'-end RACE results indicate that the NM_001831.2 sequence is not present in colon carcinoma cells and this observation is further supported by the failure to detect the full NM_001831.2 sequence by RT-PCR in normal and cancerous colon tissue samples. Instead, both RT-PCR[12] and real time PCR can successfully detect a transcript, CLU34, which is shorter at the 5'-end and matches the most predominant CLU mRNA isoform previously reported[21,2]. This shorter sequence has implications for the nature of the predicted protein because the ATG of the RefSeq NM_001831.2 exon 1 is not part of the CLU34 transcript and consequently the ORF of the predicted protein starts in exon 2. Hence identical proteins are predicted from the CLU35 and CLU34 transcripts. Importantly, this common protein is predicted to be secreted which fits exactly with what we find in our model system, as concomitantly with the induction of CLU34 mRNA an abrupt increase in cytoplasmic and secreted CLU protein species is observed. Thus, the CLU34 mRNA species apparently produce these protein variants.

The existence of two human CLU transcripts (CLU34/35) probably encoding identical CLU proteins is paralleled by previous findings in quail which demonstrated that two CLU transcripts with different, mutually exclusive, non-coding 5' exons can be produced from the avian gene. However, whereas the avian CLU transcripts are produced from two promoters often being co-regulated as suggested by correlating expression levels of the two CLU transcripts in tissue samples from a variety of organs[11], the human CLU transcripts are clearly differentially regulated.

## Conclusion

In conclusion, we have demonstrated that the Wnt signaling pathway specifically regulates one out of three CLU mRNA variants via TCF1. This CLU transcript is shorter at the 5' end than reported by the RefSeq database, and produces the intracellular 60 kDa CLU protein isoform which is secreted as a ~80 kDa protein after post-translational processing.

## Methods

### Cell culture and transient transfections

LS174T derived and HCT116 cell lines were maintained in RPMI 1640 + 25 mM hepes medium (Invitrogen/Gibco, Carlsbad, CA, USA) and McCoy's 5A medium, respectively. Each medium was supplemented with 5% foetal bovine serum (Invitrogen/Gibco) and 1% penicillin-streptomycin (Invitrogen/Gibco).

The inducible LS174T derived cell lines have previously been described[20] and were a kind gift from Dr. Hans Clevers (The Hubrecht Laboratory, The Netherlands). Induction was performed using 1 μg/ml doxycycline (Invitrogen). Selection was performed on all the LS174T derived cell lines using 10 μg/ml blasticidin (Invitrogen). 500 μg/ml zeocin (Invitrogen) was additionally used for inducible LS174T dnTCF1/4 cell lines. All cell lines were free of mycoplasma contamination as verified by the MycoSensor™ PCR assay kit (Stratagene, La Jolla, CA, USA) according to manufacturers instructions.

For transient transfection experiments involving immunofluorescence, LS174T and HCT116 colon carcinoma cell lines were transfected using Fugene 6 (Roche Applied Science, Hvidovre, DK), according to the instructions of the supplier. For transient transfection experiments involving western blotting, LS174T cells were resuspended at 2 × 10^6 ^cells/transfection in Amaxa buffer (solution V) and electroporated (program T30) with 10 μg of DNA per construct as per manufacturer's protocol (Amaxa, Cologne, Germany) and grown in 6-well plates. The GFP-cyt-E-cadherin plasmid was a kind gift from Dr. Philippe Blache (Institut de Génétique Humaine, Centre National de la Recherche Scientifique, France) and has previously been described[33]. The GFP control plasmid, kindly provided by ph.d Sanne Harder Olesen, was a pcDNA3.1 vector harboring the EGFP gene excised from the pEGFP-N1 vector (Clontech).

### Cell death assays

Two cell death assays, based on either trypan blue or propidium iodide (PI; Invitrogen) and hoechst 342 (HST; Invitrogen), were used to assess cell death rate in LS174T dnTCF1 and control cell lines 24 and 48 hr after induction. In brief, preattached cells were induced, and total number of cells (floating and attached) were pelleted. Cells were resuspended in either trypan blue solution (Sigma-Aldrich A/S, Copenhagen, Denmark) or a PBS solution containing DNA intercalating dyes, PI (10 μg/ml) and HST (20 μg/ml), and aliquots were transferred to a haemocytometer. For the trypan blue dye exclusion assay, cells were observed under a light microscope, and the extent of cell death was calculated as the percentage of stained cells (apoptotic and/or necrotic) relative to total cell number. For the PI/HST assay, images of stained nuclei were captured using a camera with appropriate filters mounted on a conventional fluorescence microscope. Viable cells were identified by intact nuclei with blue HST fluorescence and necrotic/apoptotic cells (permeable to PI) were identified by red PI fluorescence. To define a fixed area, accompanying white light (phase) pictures revealing the haemocytometer grid were layered on top of the fluorescence pictures in Adobe Photoshop software. An unpaired student's t-test was used to determine significant differences. Both assays were performed in biological triplicates.

### Proliferation and viability assays

Proliferation rate of uninduced and induced LS174T derived cell lines were determined qualitatively by staining cultured cells with methyl violet (Bie & Berntsen, Rødovre, DK) after 5 days and quantitatively by manually counting attached cells in a haemocytometer after 4 days. Both assays were done in biological triplicates. Images of cells stained by methyl violet were acquired using a regular flatbed scanner after washing cells twice in PBS.

Cell viability was assessed using the Cell Proliferation Kit I (MTT) from Sigma-Aldrich according to manufacturers instructions. LS174T dnTCF1 and control cell lines were cultured in 96-well plates in medium with or without doxycycline. After 48 hours, absorbance of solubilized MTT formazan crystals was measured on a microplatereader (Labsystems Multiscan^® ^MCC/340) at 540 nm. Experiments were done in quaduplicates.

### 5'end rapid amplification of cDNA ends

5'-end RACE was performed using the Marathon-Ready™ colon adenocarcinoma cDNA library (Clontech) according to the manufacturers recommendations. 5'-end RACE products were generated using an antisense CLU gene specific 5'-RACE primer complementary to part of CLU exon 3: 5'- GGCATCCTCTTTCTTCTTCTTGGCTTC-3'. The Marathon RACE PCR reaction was performed with Advantage 2 Polymerase Mix (50×) (Clontech). PCR products were gel purified using the QIAquick Gel Extraction Kit (Qiagen), and cloned using the TOPO TA Cloning^® ^Kit for Sequencing (Invitrogen) according to the manufacturers instructions. Cloned products from three colonies were sequenced using Expand High Fidelity PCR System (Roche Applied Science) and BigDye Terminator Kit (Applied Biosystems) with an ABI 3100 Genetic Analyzer (Applied Biosystems). The following plasmid specific internal primers were used for sequencing; 5'-TAATACGACTCACTATAGGG-3' (sense) and 5'-CAGGAAACAGCTATGAC-3' (reverse)

### Western blotting

Whole cell extracts were prepared; pelleted cells were lysed on ice in a lysis buffer containing 50 mM Tris, pH 7.5, 150 mM NaCl, 1% NP-40, 0.5% deoxycholic acid supplemented with protease inhibitor cocktail (Roche Applied Science). Analysis of CLU levels in the culture medium was done by acetone precipitation of the proteins in the culture medium, after which the pellet was resuspended in lysis buffer. Protein concentrations were determined by the Bradford assay. Samples were boiled 3 min in loading buffer (350 mM Tris HCL, 30% glycerol, 0.1% SDS, 600 mM DTT, 0.012% W/V bromophenol blue) prior to loading an equal amount of protein (20 μg cell extract, 40 μg from medium) on each lane on precast NuPAGE 12% Bis-Tris Gel (Invitrogen). Electrophoresis and blotting onto polyvinylidene fluoride (PVDF) membrane were performed according to standard minigel procedures for the Novex XCell II Mini-Cell system. All-Blue prestained standards (Bio-Rad) were used as molecular weight markers. After blocking with 3% skimmed milk, membrane was incubated with primary antibodies; mouse monoclonal anti-CLU (1:400; clone 41D; Upstate, Charlottesville, VA, USA), mouse monoclonal anti-c-Myc (1:100; clone 9E10; Santa Cruz Biotechnology, Santa Cruz, CA, USA), mouse monoclonal anti-TCF-1 (1 μg/ml; clone 7H3; Upstate), and mouse monoclonal anti-TCF-4 (1 μg/ml; clone 6H5-2; Upstate). Final protein detection used the secondary HRP-conjugated polyclonal goat anti-mouse antibody (1:2500; DakoCytomation) in combination with ECL Plus reagent (Amersham Biosciences). As loading control, parallel immunoblotting using an anti-β-actin mouse monoclonal antibody (0.05 μg/ml; clone AC-15; Sigma-Aldrich) was performed.

### Immunofluorescence

For immunofluorescence analysis, cells were cultured on glass coverslips. Cells were washed with PBS, fixed with 4% paraformaldehyde for 10 min at RT after which fixation was quenched by incubating in 0.1 M glycine-PBS solution for 5 min. Cells were permeabilized for 5 min with 0.1% Triton X-100 in PBS, washed with PBS, and blocked with 1% BSA in PBS for 45 min at 37°C. After washing with PBS, cells were incubated with primary antibodies, mouse monoclonal anti-CLU (1:400; clone 41D; Upstate) and mouse monoclonal anti-β-catenin (1:200; BD Transduction Laboratories), followed by another wash with PBS and then incubated with secondary antibody, AlexaFlour 546 goat anti-mouse IgG1 (1:400; Molecular Probes Inc.), for 1 h at 37°C. After being washed with PBS, nuclei was stained using DAPI (4,6-diamidino-2-phenylindole) (Sigma-Aldrich Denmark A/S, Copenhagen, DK), and cells were mounted on microscope slides and images were captured using a CCD camera (Quantix, Photometrics, Tucson, AZ, USA) mounted on a Leica DMRXA epifluorescence microscope (Leica, Wetzlar, Germany) with appropriate filters and operated via SmartCapture software (Digital Scientific, Cambridge, UK).

### Real time RT-PCR

Quantitative real time RT-PCR was performed on an ABI PRISM^® ^7000 Sequence Detection System (Applied Biosystems, Foster City, CA, USA) using the relevant (TaqMan or SYBR Green) Master Mix (Applied Biosystems). For normalization, the gene Ubiquitin C (UBC) was used using primer sequences previously published[34]. Total RNA was purified using the GenElute™ Mammalian total RNA miniprep kit (Sigma-Aldrich), including on-column DNase digestion (Qiagen). RNA integrity was evaluated by Bioanalyzer analysis using nano chips (Agilent Technologies). cDNA was generated using the Superscript™ cDNA synthesis kit (Invitrogen) with random nonamer primers and RNAse inhibitor (Ambion, Austin, TX, USA). The individual CLU mRNA variants were quantified using variant specific primer/probe sets previously reported[12]. Each measurement was performed in triplicate, and no-template controls were included for each assay. Relative expression values were obtained using a four-point 10-fold dilution curve. The dilution curve was created using a cDNA pool containing 2 μl of each of the test cDNAs.

### Sequencing of the genomic CLU locus

The integrity of the genomic CLU locus was analyzed by bi-directional sequencing using the BigDye Terminator Kit (Applied Biosystems) and the ABI 3100 genetic analyzer (Applied Biosystems). Fragments representing each exon and the adjacent intron-exon boundaries were generated by PCR. Primer details of the sense and antisense primers have been published elsewhere[12]. Sequencing covered all 11 exons (Fig. 5A,B; 1a, 1b, 1c, and 2–9).

## Competing interests

The author(s) declare that they have no competing interests.

## Authors' contributions

TS carried out the experiments. FM performed 5'-end RACE. CLA participated in experimental design and interpretation. TS and CLA scored transiently transfected cells and drafted the manuscript. LLC assisted in transient transfection experiments. TFØ participated in the design and coordination of the study. All authors read and approved the final manuscript.

## Supplementary Material

Additional File 1 **Quantitative examination of CLU-positive cells**. HCT116 and LS174T cells were transfected with GFP-cyt-E-cadherin or GFP control vector. Cells were fixed and immunostained with an anti-CLU antibody 24 hr after transfection. >150 transfected (green) cells were counted and scored independently for CLU (red) up-regulation by two observers, TS and CLA. Click here for file

Additional File 2 **Basal CLU expression in various prostate and colon carcinoma cell lines**. Real time RT-PCR was used to measure basal CLU expression levels of three CLU mRNA variants in various prostate and colon carcinoma cell lines. Expression levels were normalized to the Ubiquitin C (UBC) transcript. CLU34 levels varied highly across the entire panel of cell lines, whereas CLU35 and CLU36 were consistently expressed at very low levels, in some samples below the limits of detection by the real time RT-PCR assay. Click here for file

## References

[B1] Fritz IB, Burdzy K, Setchell B, Blaschuk O (1983). Ram rete testis fluid contains a protein (clusterin) which influences cell-cell interactions in vitro. Biol Reprod.

[B2] Trougakos IP, Gonos ES (2002). Clusterin/apolipoprotein J in human aging and cancer. Int J Biochem Cell Biol.

[B3] Shannan B, Seifert M, Leskov K, Willis J, Boothman D, Tilgen W, Reichrath J (2006). Challenge and promise: roles for clusterin in pathogenesis, progression and therapy of cancer. Cell Death Differ.

[B4] Zhang H, Kim JK, Edwards CA, Xu Z, Taichman R, Wang CY (2005). Clusterin inhibits apoptosis by interacting with activated Bax. Nat Cell Biol.

[B5] Leskov KS, Klokov DY, Li J, Kinsella TJ, Boothman DA (2003). Synthesis and functional analyses of nuclear clusterin, a cell death protein. J Biol Chem.

[B6] Criswell T, Beman M, Araki S, Leskov K, Cataldo E, Mayo LD, Boothman DA (2005). Delayed activation of insulin-like growth factor-1 receptor/Src/MAPK/Egr-1 signaling regulates clusterin expression, a pro-survival factor. J Biol Chem.

[B7] Jin G, Howe PH (1999). Transforming growth factor beta regulates clusterin gene expression via modulation of transcription factor c-Fos. Eur J Biochem.

[B8] Loison F, Debure L, Nizard P, le Goff P, Michel D, le Drean Y (2006). Up-regulation of the clusterin gene after proteotoxic stress: implication of HSF1-HSF2 heterocomplexes. Biochem J.

[B9] Suh E, Wang Z, Swain GP, Tenniswood M, Traber PG (2001). Clusterin gene transcription is activated by caudal-related homeobox genes in intestinal epithelium. Am J Physiol Gastrointest Liver Physiol.

[B10] Cervellera M, Raschella G, Santilli G, Tanno B, Ventura A, Mancini C, Sevignani C, Calabretta B, Sala A (2000). Direct transactivation of the anti-apoptotic gene apolipoprotein J (clusterin) by B-MYB. J Biol Chem.

[B11] Michel D, Chatelain G, Herault Y, Brun G (1995). The expression of the avian clusterin gene can be driven by two alternative promoters with distinct regulatory elements. Eur J Biochem.

[B12] Andersen CL, Schepeler T, Thorsen K, Birkenkamp-Demtroder K, Mansilla F, Aaltonen LA, Laurberg S, Orntoft TF (2007). Clusterin expression in normal mucosa and colorectal cancer. Mol Cell Proteomics.

[B13] Cochrane DR, Wang Z, Muramaki M, Gleave ME, Nelson CC (2007). Differential regulation of clusterin and its isoforms by androgens in prostate cells. J Biol Chem.

[B14] Thomas-Tikhonenko A, Viard-Leveugle I, Dews M, Wehrli P, Sevignani C, Yu D, Ricci S, el-Deiry W, Aronow B, Kaya G, Saurat JH, French LE (2004). Myc-transformed epithelial cells down-regulate clusterin, which inhibits their growth in vitro and carcinogenesis in vivo. Cancer Res.

[B15] He TC, Sparks AB, Rago C, Hermeking H, Zawel L, da Costa LT, Morin PJ, Vogelstein B, Kinzler KW (1998). Identification of c-MYC as a target of the APC pathway. Science.

[B16] Chen T, Turner J, McCarthy S, Scaltriti M, Bettuzzi S, Yeatman TJ (2004). Clusterin-mediated apoptosis is regulated by adenomatous polyposis coli and is p21 dependent but p53 independent. Cancer Res.

[B17] Burkey BF, deSilva HV, Harmony JA (1991). Intracellular processing of apolipoprotein J precursor to the mature heterodimer. J Lipid Res.

[B18] Nizard P, Tetley S, Le Drean Y, Watrin T, Le Goff P, Wilson MR, Michel D (2007). Stress-Induced Retrotranslocation of Clusterin/ApoJ into the Cytosol. Traffic.

[B19] Burkey BF, Stuart WD, Harmony JA (1992). Hepatic apolipoprotein J is secreted as a lipoprotein. J Lipid Res.

[B20] van de Wetering M, Sancho E, Verweij C, de Lau W, Oving I, Hurlstone A, van der Horn K, Batlle E, Coudreuse D, Haramis AP, Tjon-Pon-Fong M, Moerer P, van den Born M, Soete G, Pals S, Eilers M, Medema R, Clevers H (2002). The beta-catenin/TCF-4 complex imposes a crypt progenitor phenotype on colorectal cancer cells. Cell.

[B21] Wong P, Taillefer D, Lakins J, Pineault J, Chader G, Tenniswood M (1994). Molecular characterization of human TRPM-2/clusterin, a gene associated with sperm maturation, apoptosis and neurodegeneration. Eur J Biochem.

[B22] UCSC Genome Browser. http://genome.ucsc.edu/index.html.

[B23] PSORT II. http://psort.ims.u-tokyo.ac.jp.

[B24] Gleave M, Miyake H (2005). Use of antisense oligonucleotides targeting the cytoprotective gene, clusterin, to enhance androgen- and chemo-sensitivity in prostate cancer. World J Urol.

[B25] Steinberg J, Oyasu R, Lang S, Sintich S, Rademaker A, Lee C, Kozlowski JM, Sensibar JA (1997). Intracellular levels of SGP-2 (Clusterin) correlate with tumor grade in prostate cancer. Clin Cancer Res.

[B26] MatInspector software. http://www.genomatix.de.

[B27] Patel NV, Wei M, Wong A, Finch CE, Morgan TE (2004). Progressive changes in regulation of apolipoproteins E and J in glial cultures during postnatal development and aging. Neurosci Lett.

[B28] Van Lenten BJ, Wagner AC, Navab M, Fogelman AM (2001). Oxidized phospholipids induce changes in hepatic paraoxonase and ApoJ but not monocyte chemoattractant protein-1 via interleukin-6. J Biol Chem.

[B29] Pinto D, Clevers H (2005). Wnt, stem cells and cancer in the intestine. Biol Cell.

[B30] French LE, Chonn A, Ducrest D, Baumann B, Belin D, Wohlwend A, Kiss JZ, Sappino AP, Tschopp J, Schifferli JA (1993). Murine clusterin: molecular cloning and mRNA localization of a gene associated with epithelial differentiation processes during embryogenesis. J Cell Biol.

[B31] Klock G, Storch S, Rickert J, Gutacker C, Koch-Brandt C (1998). Differential regulation of the clusterin gene by Ha-ras and c-myc oncogenes and during apoptosis. J Cell Physiol.

[B32] Tice DA, Soloviev I, Polakis P (2002). Activation of the Wnt pathway interferes with serum response element-driven transcription of immediate early genes. J Biol Chem.

[B33] Blache P, van de Wetering M, Duluc I, Domon C, Berta P, Freund JN, Clevers H, Jay P (2004). SOX9 is an intestine crypt transcription factor, is regulated by the Wnt pathway, and represses the CDX2 and MUC2 genes. J Cell Biol.

[B34] Andersen CL, Jensen JL, Orntoft TF (2004). Normalization of real-time quantitative reverse transcription-PCR data: a model-based variance estimation approach to identify genes suited for normalization, applied to bladder and colon cancer data sets. Cancer Res.

