# Supplementary material for: Clusterin expression can be modulated by changes in TCF1-mediated Wnt signaling
Source: J Mol Signal. 2007 Jul 16;2:6. doi: 10.1186/1750-2187-2-6 (PMC1976611; doi:10.1186/1750-2187-2-6)
Supplement: Additional File 1 — Quantitative examination of CLU-positive cells. HCT116 and LS174T cells were transfected with GFP-cyt-E-cadherin or GFP control vector. Cells were fixed and immunostained with an anti-CLU antibody 24 hr after transfection. >150 transfected (green) cells were counted and scored independently for CLU (red) up-regulation by two observers, TS and CLA. [file 1750-2187-2-6-S1.pdf]

Cell line	Observer	Vector	# transfected	# CLU positive	% CLU positive
LS174T	CLA	GFP-cyt-E-cad	158	48	30
		GFP control	159	13	8
	TS	GFP-cyt-E-cad	157	47	30
		GFP control	156	21	13
HCT116	CLA	GFP-cyt-E-cad	151	67	44
		GFP control	150	11	7
	TS	GFP-cyt-E-cad	152	57	38
		GFP control	151	10	7
