# Supplementary material for: Clusterin expression can be modulated by changes in TCF1-mediated Wnt signaling
Source: J Mol Signal. 2007 Jul 16;2:6. doi: 10.1186/1750-2187-2-6 (PMC1976611; doi:10.1186/1750-2187-2-6)
Supplement: Additional File 2 — Basal CLU expression in various prostate and colon carcinoma cell lines. Real time RT-PCR was used to measure basal CLU expression levels of three CLU mRNA variants in various prostate and colon carcinoma cell lines. Expression levels were normalized to the Ubiquitin C (UBC) transcript. CLU34 levels varied highly across the entire panel of cell lines, whereas CLU35 and CLU36 were consistently expressed at very low levels, in some samples below the limits of detection by the real time RT-PCR assay. [file 1750-2187-2-6-S2.pdf]

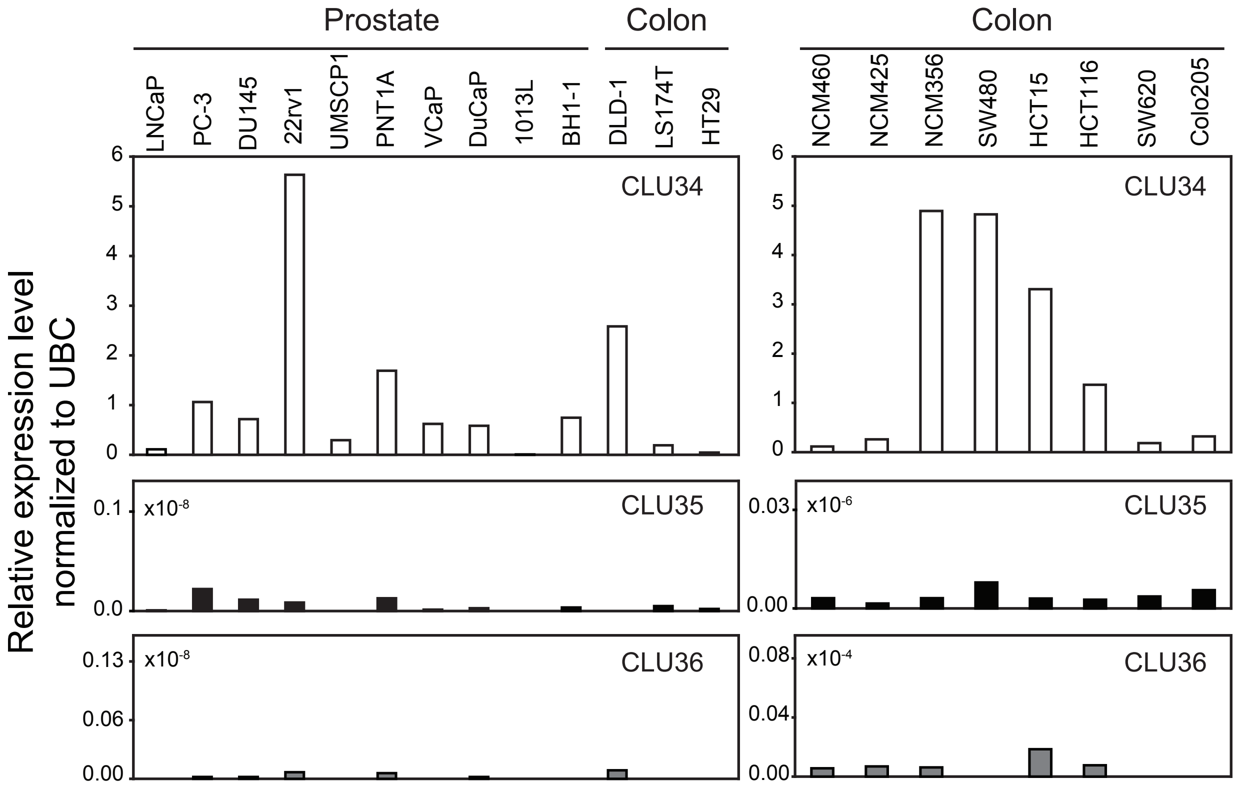
Prostate
Colon
Colon
LNCaP
PC-3
DU145
22rv1
UMSCP1
PNT1A
VCaP
DuCaP
1013L
BH1-1
DLD-1
LS174T
HT29
NCM460
NCM425
NCM356
SW480
HCT15
HCT116
SW620
Colo205
Relative expression level normalized to UBC
CLU34
CLU35
CLU36
x10-8
x10-6
x10-4
6
5
4
3
2
1
0
0.1
0.0
0.03
0.00
0.13
0.06
0.00
0.08
0.04
0.00
